# Evidence map of Tai Chi interventions for older adults

**DOI:** 10.3389/fpubh.2026.1820294

**Published:** 2026-07-02

**Authors:** Lissandra Zanovelo Fogaça, Mariana Cabral Schveitzer, Arthur Ferreira, Andreia do Carmo Feitosa, Michael Pratt, Caio Fábio Schlechta Portella, Ricardo Ghelman, Luiz Roberto Ramos

**Affiliations:** 1 Department of Preventive Medicine, Paulista School of Medicine, Federal University of São Paulo, São Paulo, Brazil; 2 University Center of Jales—UNIJALES, Jales, Brazil; 3 University of California, San Diego, San Diego, CA, United States; 4 Brazilian Academic Consortium for Integrative Health—CABSIN, São Paulo, Brazil

**Keywords:** complementary therapies, evidence map, older adults, public health, Tai Chi

## Abstract

The population aged 60 and over is growing rapidly worldwide, reflecting rising life expectancy and health challenges. Tai Chi is a Chinese mind–body practice and has been recommended for older adults. Evidence maps organize and synthesize information about evidence in a specific field or topic, highlighting consensus and knowledge gaps. This evidence map aims to identify the effects of Tai Chi on health outcomes in older adults. This map used The Campbell Collaboration’s methodology. The search was conducted in five databases: PubMed, Embase, CINAHL, Scopus, and Web of Science. The Brazilian Academic Consortium for Integrative Health data of Traditional Chinese Medicine mind–body practices was utilized to manually include studies. The AMSTAR 2 was used to assess quality assessment, and the Tableau was used to graphically present the results. Based on 7.862 systematic reviews, we included 118 of Tai Chi for older adults. The quality assessment was 36 high, 07 moderate, 42 low, and 33 critically low. Each of the 238 effects was classified: 171 as positive, 47 as potentially positive, 13 no effects, and 07 as inconclusive. High-quality evidence that positive effects were related to balance, depression, anxiety, cognitive performance, risk of fall, quality of life, well-being, sleep quality, and Parkinson’s disease. Inconclusive or no effect was related to concentration, agility, muscle mass, HDL cholesterol, stroke, hypertension, and coronary heart disease. The most widely used Tai Chi style was the Yang style, for two to three times per week, with each session lasting 30 to 60 min for 12 to 24 weeks. Most interventions targeted community-dwelling older adults with a mean age of 69.5 years; among these, 42 reviews reported a female majority. Based on 56 associations, Tai Chi has been applied in different areas of older adult health. The effect of tai chi may depend on the style, frequency, time, and duration. Despite all results, the evidence suggests methodological rigor, homogeneous populations with larger samples. The Tai Chi effectiveness evidence map will serve to inform decision-making on healthcare for older adults, considering the Decade of Healthy Ageing 2021–2030 and the Global Strategy for Traditional, Complementary, and Integrative Medicine 2025–2034.

## 1 Introduction

The number of people aged 60 and over is expected to reach 1.4 billion by 2030 and 2.1 billion by 2050, reflecting rising life expectancy and presenting challenges, particularly in public health, the economy, and social support. Nations will need to prepare by strengthening health and long-term care systems and ensuring the sustainability of social protection (1, 2). Recently, the World Health Organization (WHO) endorsed a global strategy on aging and health that outlines a plan for a Decade of Healthy Aging 2020–2030 that urges countries to take action to ensure that all individuals can live long and healthy lives, and emphasizes the importance of action to support healthy aging improved three basics components functional ability, intrinsic capacity (IC) and environments (3, 4).

Functional ability is composed of a combination of IC with the environment and the integration of both. On the other hand, IC is the indicator that combines physical and mental capabilities that considers the integration of locomotor, vitality, cognitive, psychological, and sensory abilities (5, 6). Higher IC levels are associated with a reduced risk of disability and better overall quality of life, especially in older adults (7). To address this, health care needs to recognize and manage the decline in intrinsic capacity and maintain functional capacity by providing holistic and integrated care that responds to the diverse health and care needs of older adults (1).

WHO has been encouraging and strengthening the use of traditional, complementary, and integrative medicines (TCIM), products, therapies, and their practitioners at all levels of healthcare activity, through the recommendations of the WHO Strategy on Traditional Medicine. The strategy aims to promote the integration of safe and effective TCIM into health systems in evidence-based ways, holistic, centred care for people, empower communities, be culturally respectful, and align with sustainable development (8–11). TCIM is an important and valuable health resource with many applications, especially in the prevention and management of lifestyle-related chronic diseases, and in meeting the health needs of ageing populations (12).

Tai Chi (TC) is an oriental mind–body practice and a form of TCIM considered a light-to-moderate intensity exercise that consists of a triad of breathing, meditation, and physical movement with impacts on different outcomes across multiple physiological and health conditions (13–15). The practice of TC has effects on balance, rate of falls, risk of falls, cognitive performance, memory, visuospatial capacity, and the prevention of falls in aged people (16). Currently, increasing evidence shows that TC practice could significantly enhance musculoskeletal disorders (17), diverse chronic diseases (18), reduce depression and anxiety symptoms, and improve quality of life (QOL) (19, 20). In particular, researchers recommend Tai Chi to promote healthy aging among nursing home residents (21).

Evidence and Gap Maps (EGMs) are tools used to organize, synthesize, and visually display existing research to support evidence-informed decision-making for researchers, commissioners, and policymakers. They compile evidence on intervention effects and related outcomes within a defined thematic area, typically arranged in an interactive matrix that shows where evidence is available and where research gaps remain (22).

This evidence map aims to identify and synthesize the effect of TC on health outcomes in older adults’ care, and report quality assessment of systematic reviews. In addition the map identifies and reports on the settings in which the studies were conducted, the older adult age ranges, health diversity of study populations, TC intervention style, intervention durations, and frequency of weekly/yearly classes. Despite growing evidence, no comprehensive evidence map has synthesized Tai Chi effects across all older-adult health domains or identified style-specific or regional (e.g., Tai Chi Pai Lin) gaps. In this EGM, we also aim to establish a connection between population characteristics, reported health outcomes, and key features of the TC interventions, and present this in as concise and structured a way as possible.

## 2 Method

Evidence and Gap Maps (EGMs) are a tool designed to synthesize and visually present existing research evidence to support evidence-informed decision-making for researchers, commissioners, and decision makers. EGMs provide thematic collections of evidence on effects structured around interventions and outcomes of relevance to a particular thematic area, typically displayed as an interactive matrix, highlighting what is known and identifying “gaps” in research that are lacking (23).

The guide to produce an EGM uses a PICOS framework, a comprehensive search on databases, screening of studies against explicit inclusion and exclusion criteria, coding, analysis, and outcome synthesis (24). Moreover, an overall quality assessment of pertinent evidence is characterized and made accessible (25, 26).

This EGM, developed in accordance with the Campbell Collaboration methodological guidelines, systematically synthesizes the available interventions and associated health outcomes of Tai Chi for older adults. The development process encompassed six structured and sequential stages: (1) comprehensive literature search, (2) study selection based on predefined eligibility criteria, (3) thematic and methodological categorization, (4) informetric analysis, (5) construction of the evidence map, and (6) identification and characterization of gaps (27). All methodological procedures and the corresponding results were reported in accordance with the PRISMA guidelines (28) and the Campbell Collaboration Evidence Gap Map methodology (24).

### 2.1 Data sources

Using the PICOS framework, we defined the inclusion criteria as follows: the (P) population consisted of older adults, the (I) intervention was Tai Chi, (C) no comparator was required, the (O) outcomes were health-related measures, and the (S) study designs were systematic reviews (with or without meta-analyses) and meta-analyses. These criteria guided a search across five databases as PubMed (National Library of Medicine), Embase (Elsevier), CINAHL (EBSCOhost), Scopus (Elsevier), and Web of Science (Clarivate Analytics), from inception to June 2025, including studies published in Portuguese, English, and Spanish. The Tai Chi search strategy was adapted from Ng et al. (29), and the terms for identifying older adult populations were based on van de Glind et al. (30), as detailed in Supplementary Table 1. Additionally, records from the Brazilian Academic Consortium for Integrative Health (CABSIN) on Traditional Chinese Medicine Mind–Body Practices were consulted to manually complement the dataset.

### 2.2 Inclusion and exclusion criteria

The inclusion criteria were: systematic reviews with or without meta-analyses with at least two primary studies that examined Tai Chi interventions and reported health-related outcomes, and studies in which the population consisted of older adults aged 60 years or older, irrespective of health status. We excluded systematic reviews in which Tai Chi was combined with another intervention in a way that prevented isolated assessment of Tai Chi effects, as well as reviews that focused on other mind–body practices. Review protocols were also excluded.

### 2.3 Procedure

#### 2.3.1 Screening and selection

Two independent reviewers, working in a blinded manner, screened all search results using Rayyan. Records judged as potentially relevant by either reviewer, as well as those for which relevance could not be determined, were retrieved in full text. Full-text articles were assessed against the predefined inclusion criteria by the same pair of reviewers. Disagreements were resolved through discussion until a consensus was reached. The full screening and selection process is presented in the PRISMA-ScR flow diagram.

#### 2.3.2 Data extraction

A characterization matrix in Supplementary Table 2 was developed to extract and organize these findings. This matrix contained specific fields such as Title, Number total of population (as described by reviewer), Age range/age, Population description (as described by reviewer—e.g., older adults with Parkinson’s disease, older adults with sleep disorders), Setting (local—e.g., community, long-term care centers), Intervention details (duration in days/weeks/months; weekly frequency, and minutes/h per day), Tai Chi style (e.g., Tai Chi Unspecified, Yang style), Outcomes Groups (e.g., Physical Indicators, Chronic Diseases), Outcomes (e.g., depression, balance), Effects (e.g., positive, potential positive), Adverse event available (e.g., Adverse event not available, No adverse event), Adverse event (e.g., pain, fatigue), Journal, Quality assessment (e.g., high, moderate, low), Type of Review, Study Design, Study Focus, Focus Country, Publication Country, Publication Year, Citation, and Link to full texts (when available). All extracted information was subsequently integrated into the interactive Tableau platform to enable the bubble plot graphical presentation of the evidence and facilitate exploration of the mapped findings.

#### 2.3.3 Quality assessment tool

We assessed the methodological quality of the included systematic reviews using AMSTAR 2 (*Assessing the Methodological Quality of Systematic Reviews*). This tool consists of 16 items and classifies reviews as high, moderate, low, or critically low in quality. AMSTAR 2 provides an indication of the level of confidence that can be placed in each review’s findings and highlights potential sources of bias, including selection, measurement, and confounding (31). The AMSTAR 2 assessment tool, along with its specific criteria for classification and interpretation, is available in Supplementary Material S1, S2.

#### 2.3.4 Data synthesis

The synthesis of evidence in this map followed a dual-criteria approach: (1) the intervention effect was defined by the statistical significance (*p* < 0.05) and directionality reported in the primary systematic reviews, and (2) quality assessment (methodological confidence) was determined by the AMSTAR 2 rating. A ‘Positive’ effect was only attributed when supported by statistically significant data. The AMSTAR 2 rating was then superimposed to indicate the degree of confidence the reader should have in that specific systematic review’s process. This integration ensures that practitioners can identify not only where Tai Chi works but also which findings are supported by high-quality systematic reviews and methodological standards.

To ensure fidelity to the original evidence and minimize subjective interpretation bias, the health outcomes were categorized according to the specific terminology reported in the included systematic reviews. While this results in a more granular taxonomy, it allows for a precise identification of how Tai Chi is being evaluated in current geriatric research.

A qualitative synthesis was conducted following the methodological approach recommended for EGMs. This process focused on organizing the extracted information to support the subsequent development of the evidence map. After data extraction, the characteristics of the interventions, populations, outcomes, and treatment effect classifications (positive, potential positive, inconclusive, potential negative, and negative) were systematically described. The quality assessments (AMSTAR 2) were also incorporated to ensure representation of the evidence during the mapping process. The purpose of this synthesis was to structure the available information, without performing quantitative pooling or reinterpretation of data. The analytical implications and interpretative considerations derived from this synthesis are addressed in the Discussion section of the article.

##### 2.3.4.1 Operational definition of effect labels

To ensure methodological transparency, we established explicit criteria for assigning effect labels to each Tai Chi intervention. The classification was based on a combination of statistical significance (*p* < 0.05), the direction of the effect reported in the systematic reviews, and the consistency of results across multiple reviews. The positive effect: significant improvement reported in the outcome, supported by consistent evidence across reviews. Potential positive effect: significant improvement reported in a majority of reviews, but with noted heterogeneity in primary studies or relying primarily on ‘low’ or ‘critically low’ quality reviews. Inconclusive: conflicting results across reviews (e.g., some reporting positive effects while others reported no effect) or insufficient data to determine a clear direction. No effect: no statistically significant difference reported between the Tai Chi group and the control/comparator group across the included evidence. Potential negative or negative effects: significant worsening of the outcome in the Tai Chi group; however, no such effects were identified in this map.

## 3 Results

**Figure 1:**
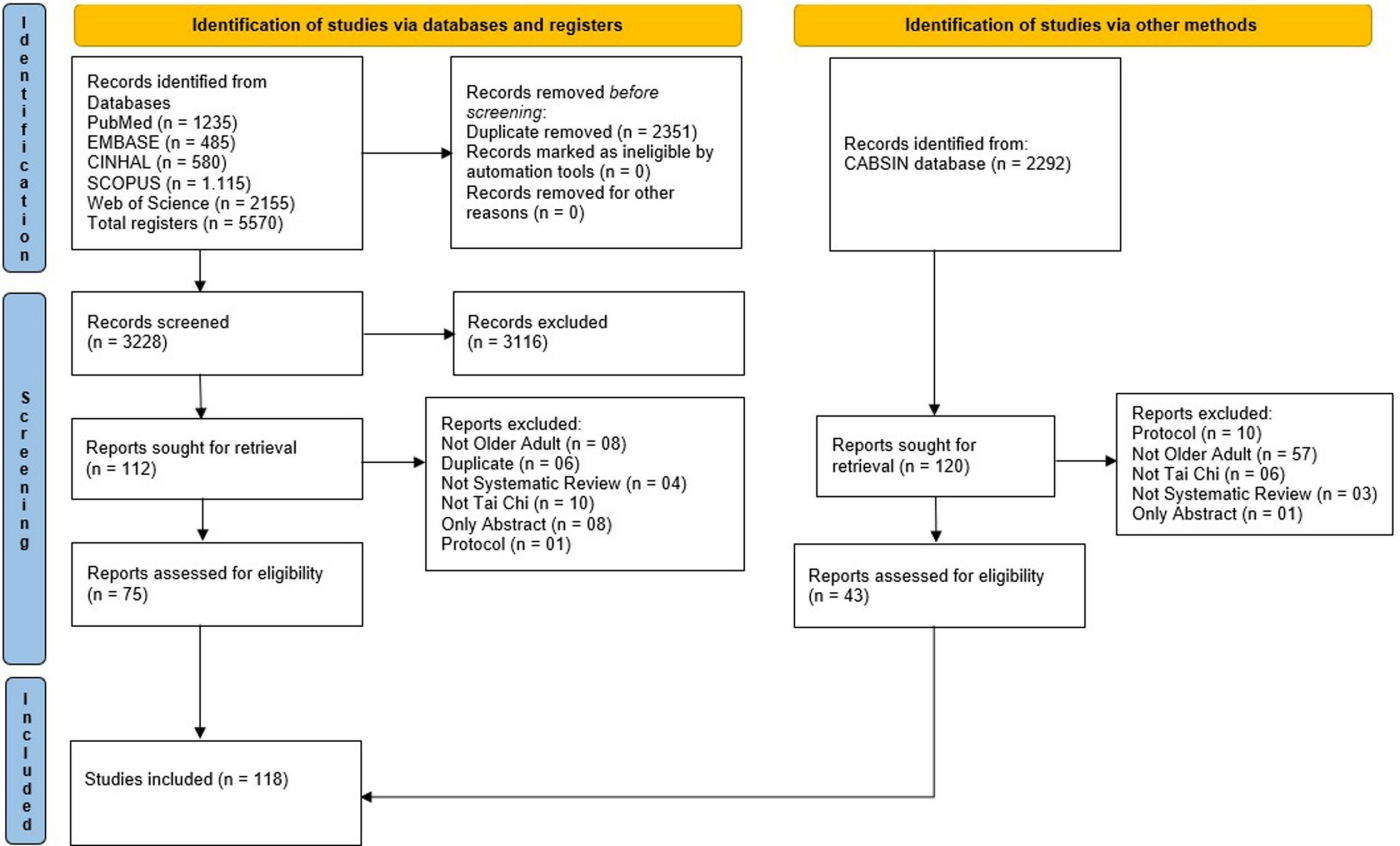
PRISMA—ScR flow diagram for new systematic reviews which included searches of databases, registers and other sources.

We identified 7.862 systematic reviews in the database search, and 118 were included (37 systematic reviews, 17 meta-analyses, 64 systematic reviews with meta-analyses) (21, 32–147) on Tai Chi for older adults. The PRISMA-ScR flow diagram shows this process (Figure 1). Most reviews have been published in the last five years.

### 3.1 Population

This evidence map analyzed data from systematic reviews about older adults and the following characteristics were identified: older adults with chronic diseases (heart disease, hypertension, osteoarthritis, cancer, chronic obstructive pulmonary disease, and diabetes), neurological/mental disorders (dementia, Parkinson’s disease, stroke, depression, anxiety, mild cognitive impairment, and sleep disorders), women in postmenopausal conditions, and older adults that have physical impairment such as sarcopenia, frailty, pre-frailty, faller, and with risk of fall. Systematic reviews exhibited heterogeneous population descriptions, frequently combining diverse subgroups within older adults, with highlighting on neurological disorders (e.g., cognitive impairment, mild cognitive impairment, and dementia), 24 reviews (53, 55, 70, 76, 84, 89, 93, 95, 98, 103, 106, 107, 110, 112, 124, 128–130, 135, 139–141, 145, 146), Parkinson’s disease that appeared in 17 reviews (40, 47, 54, 56, 60, 73, 79, 81, 85, 88, 99, 108, 110, 122, 129, 137, 140), and older adults with impaired balance (e.g., with risk of fall, faller, fear of fall, and vestibular disorders), 15 reviews (33, 38, 49, 50, 64, 71, 82, 90, 95, 100, 108, 110, 118, 140, 141). Ninety-one systematic reviews reported the total population included, which corresponds to more than 16 million older adults, considering that different systematic reviews may have used the same primary study. However, 21 systematic reviews did not report the total population. The mean older adults’ age was ~ 69.48 years. Regarding gender distribution, 42 reviews reported a female majority (more than 9 million), while seven identified a male majority, and six included both sexes. Notably, 63 reviews did not clearly specify the predominant gender across their included primary studies.

### 3.2 Settings

The settings were reported in 28 systematic reviews, with the majority of Tai Chi interventions (26) occurring in the community (community-dwelling older adults). Other systematic reviews reported different settings, such as long-term care facilities, primary health care, rehabilitation centers, and hospitals. However, eighty-nine systematic reviews did not specify the setting where the primary studies were conducted.

### 3.3 Focus country

**Figure 2:**
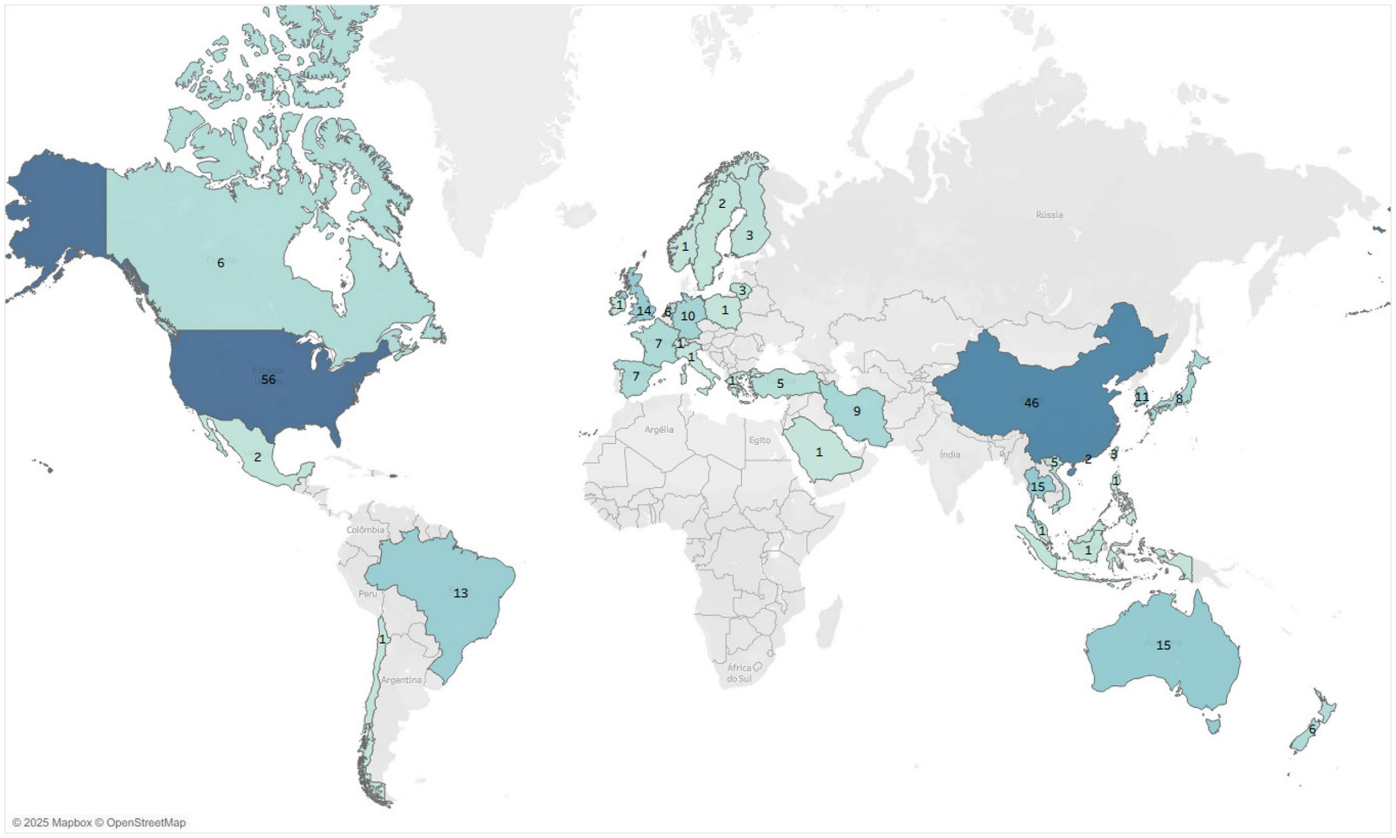
Global mapping of primary studies on Tai Chi distributed by country.

The systematic reviews included analyzed primary data from the following countries: United States of America (56), China (46), Australia (15), Thailand (15), United Kingdom (14), Brazil (13), South Korea (11), Germany (10), Iran (09), Japan (08), France (07), Spain (07), New Zealand (06), Canada (06), Netherland (06), Turkey (05), Vietnam (05), Taiwan (03), Lithuania (03), Finland (03), Sweden (02), Mexico (02), Singapore (01), Poland (1), Chile (01), Italy (01), Norway (01), Philippines (01), Ireland (1), Switzerland (01), Greece (01), and Malaysia (01), represented in Figure 2. However, the same primary study may have been used in different systematic reviews. Forty systematic reviews did not identify the primary data focus country.

### 3.4 Publication country

Brazil, Canada, China, England, Germany, Israel, Netherlands, New Zealand, Poland, Scotland, South Korea, Spain, Switzerland, the United Kingdom, and the United States of America. The United States (44), England (23), and Switzerland (16) are the countries that published the most.

### 3.5 Outcomes and effects

**Figure 3:**
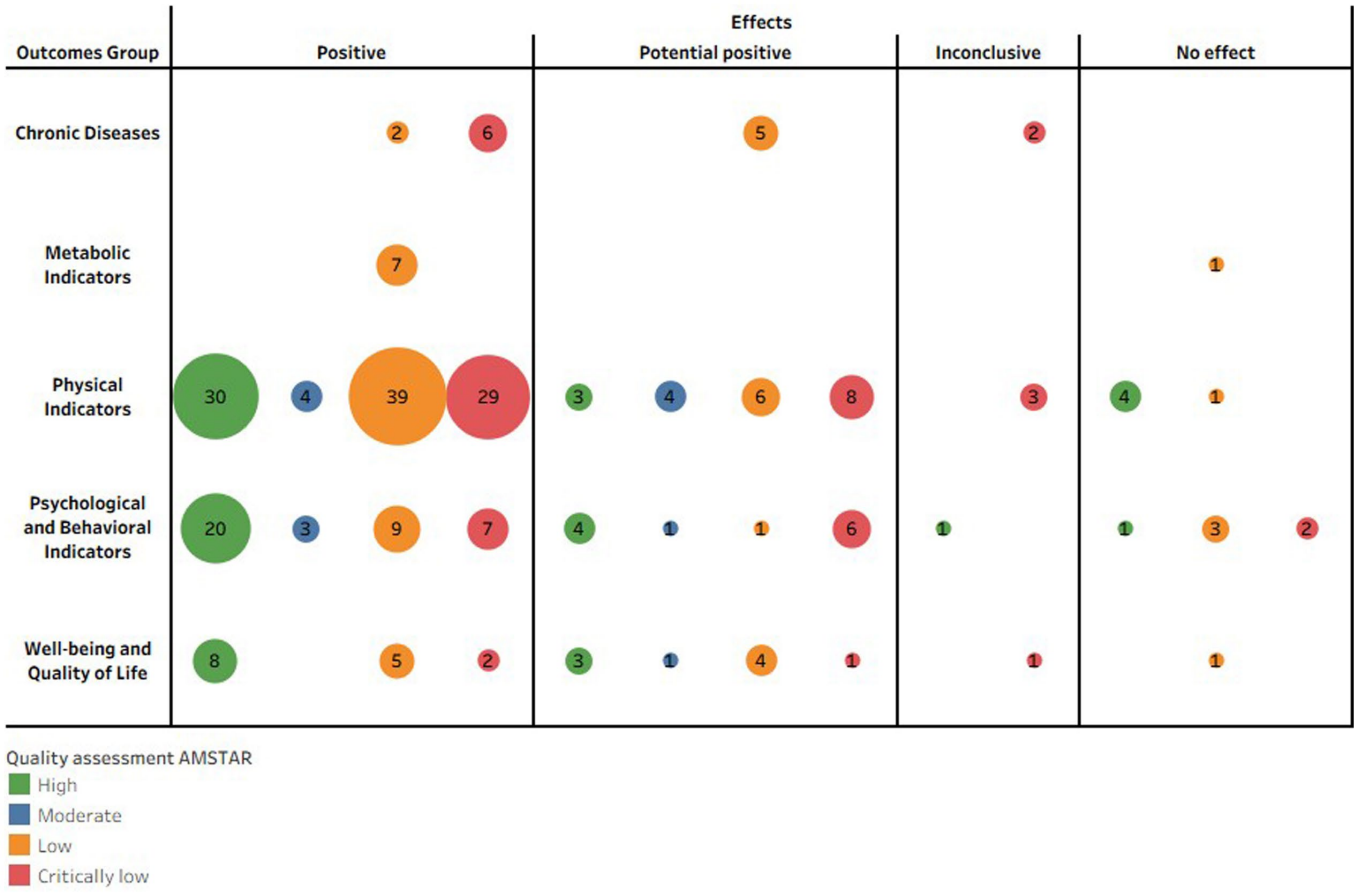
Bubble plot mapping the effectiveness of Tai Chi interventions and AMSTAR confidence levels by outcome groups category.

Tai Chi interventions for older adults’ health care were associated with 238 effects across 56 different health outcomes, classified as: 171 positive, 47 potentially positive, 13 no effect, and 07 inconclusive. Notably, no negative or potentially negative effects were identified in the analyzed literature. The results from the 118 systematic reviews were grouped into five major outcome groups: Physical Indicators (131 outcomes), Psychological and Behavioral Indicators (58), Well-being and Quality of Life (26), Chronic Diseases (15), and Metabolic Indicators (8). The outcome group, quality assessment, and effect of Tai Chi are presented in Figure 3.

### 3.6 Outcome groups

#### 3.6.1 Physical indicators

**Figure 4:**
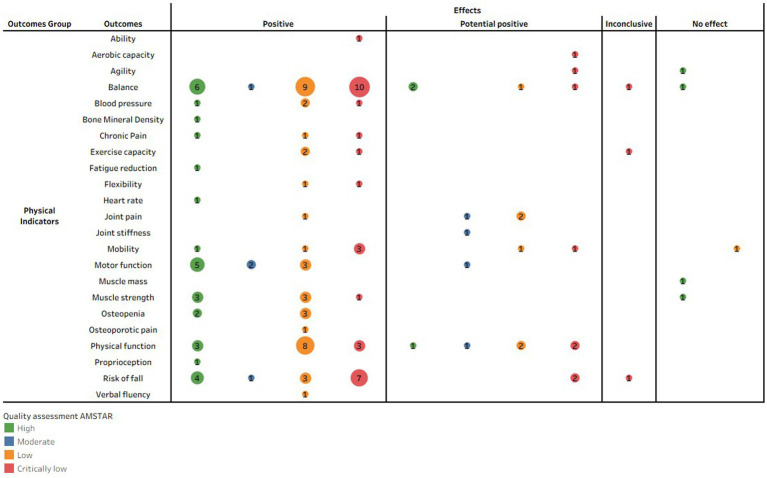
Bubble plot mapping of effectiveness and methodological quality matrix of Tai Chi interventions for specific physical indicator outcomes. Note: This breakdown highlights the specific physical metrics (e.g., balance, risk of fall), where bubble size reflects studies volume and colors represent AMSTAR confidence levels (Green = High; Blue = Moderate; Orange = Low; Red = Critically low).

One hundred and thirty-five (131) outcomes constitute the physical indicators group (Figure 4). Among them has balance, with 32 outcomes, physical function (20 outcomes), risk of fall (18), motor function (11), mobility (8), muscle strength (8), osteopenia (5), blood pressure (4), joint pain (4), exercise capacity (4), chronic pain (3), flexibility (2), ability (1), aerobic capacity (1), osteoporotic pain (1), joint stiffness (1), fatigue reduction (1), heart rate (1), proprioception (1), bone mineral density (1), muscle mass (1), and verbal fluency (1). The positive effects were highlighted related to balance (26), risk of fall (15), physical function (14), motor function (10), muscle strength (7), mobility (5), osteopenia (5), blood pressure (4), exercise capacity (3), chronic pain (3), flexibility (2), ability (1), osteoporotic pain (1), joint pain (1), fatigue reduction (1), heart rate (1), proprioception (1), bone mineral density (1), and verbal fluency (1). Potential positive outcome effects were shown in physical function (6), balance (4), joint pain (3), risk of fall (2), mobility (2), motor function (1), aerobic capacity (1), and joint stiffness (1). Inconclusive effects were related to balance (1), exercise capacity (1), and risk of fall (1). Outcomes with no effects were balance (1), agility (1), mobility (1), muscle mass (1), and muscle strength (1).

#### 3.6.2 Psychological and behavioral indicators

**Figure 5:**
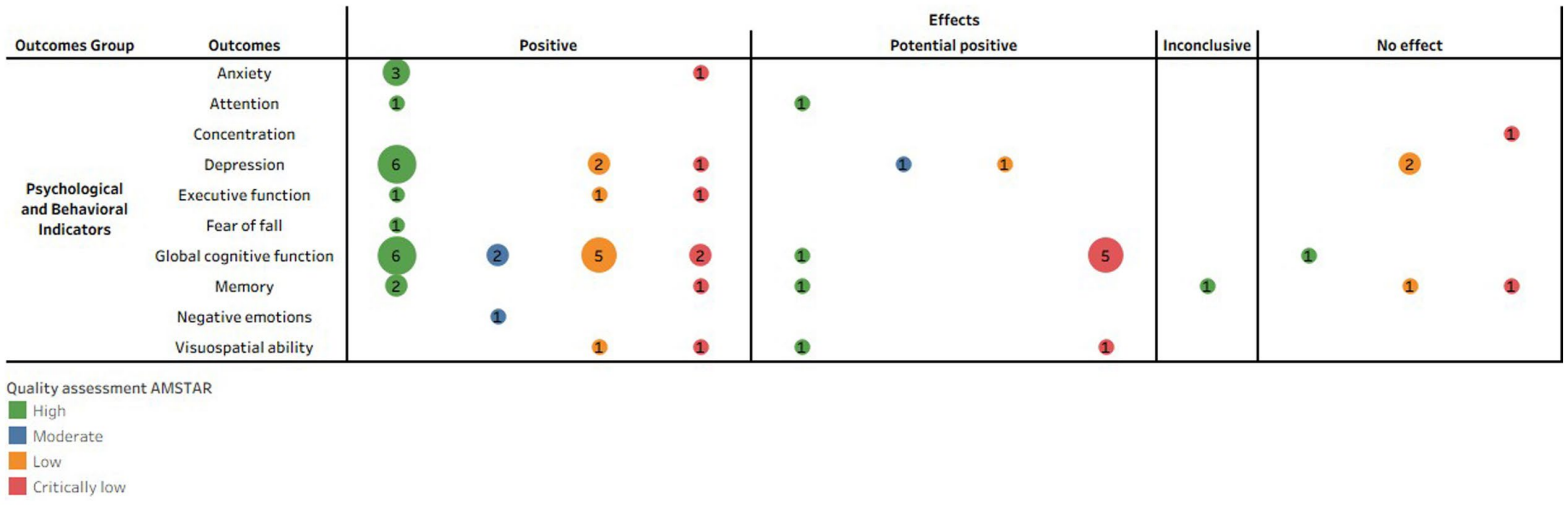
Bubble plot mapping of effectiveness and methodological quality matrix of Tai Chi interventions for specific psychological and behavioral indicator outcomes. Note: This breakdown highlights specific psychological and cognitive metrics (e.g., anxiety, depression, global cognitive function), where bubble size reflects study volume and colors represent AMSTAR confidence levels (Green = High; Blue = Moderate; Orange = Low; Red = Critically low).

The psychological and behavioral indicators in the included systematic reviews represent a total of 58 outcomes (Figure 5) and were related of global cognitive function, which appeared 22 times, depression (13), memory (7), anxiety (4), visuospatial ability (4), executive function (3), attention (2), fear of fall (1), concentration (1), and negative emotions (1). Positive effects were shown in global cognitive function (15), depression (9), anxiety (4), memory (3), executive function (3), visuospatial ability (2), attention (1), fear of fall (1), and negative emotions (1). Potential positive effects were related to global cognitive function (6), depression (2), visuospatial ability (2), attention (1), and memory (1). Inconclusive effects are related to memory (1). Outcomes related to no effect were depression (2), memory (2), concentration (1), and global cognitive function (1).

#### 3.6.3 Well-being and quality of life

**Figure 6:**
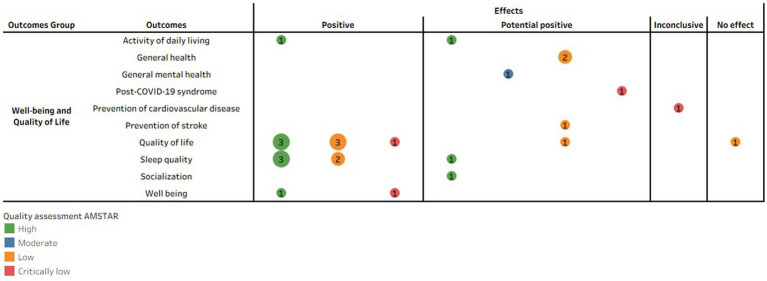
Bubble plot mapping of effectiveness and methodological quality matrix of Tai Chi interventions for specific well-being and quality of life outcomes. Note: This breakdown highlights specific holistic and functional metrics (e.g., quality of life, sleep quality, daily living activities), where bubble size reflects study volume and colors represent AMSTAR confidence levels (Green = High; Blue = Moderate; Orange = Low; Red = Critically low).

The outcomes effects on the well-being and quality of life group were a total of 26 outcomes (Figure 6) and that related to quality of life (9), sleep quality (6), well-being (2), general health (2), activity of daily living (2), socialization (1), post COVID-19 syndrome (1), prevention of stroke (1), prevention of cardiovascular disease (1), and general mental health (1). Thereby, the outcomes’ positive effects on Well-being and Quality of Life were related to quality of life (7), sleep quality (5), well-being (2), and activity of daily living (1). Potential positive effects were found in general health (2), socialization (1), activity of daily living (1), general mental health (1), post-COVID-19 syndrome (1), prevention of stroke (1), sleep quality (1), and quality of life (1). Inconclusive effects were related to the prevention of cardiovascular disease (1). We found that one outcome related to quality of life has no effect.

#### 3.6.4 Chronic diseases

**Figure 7:**
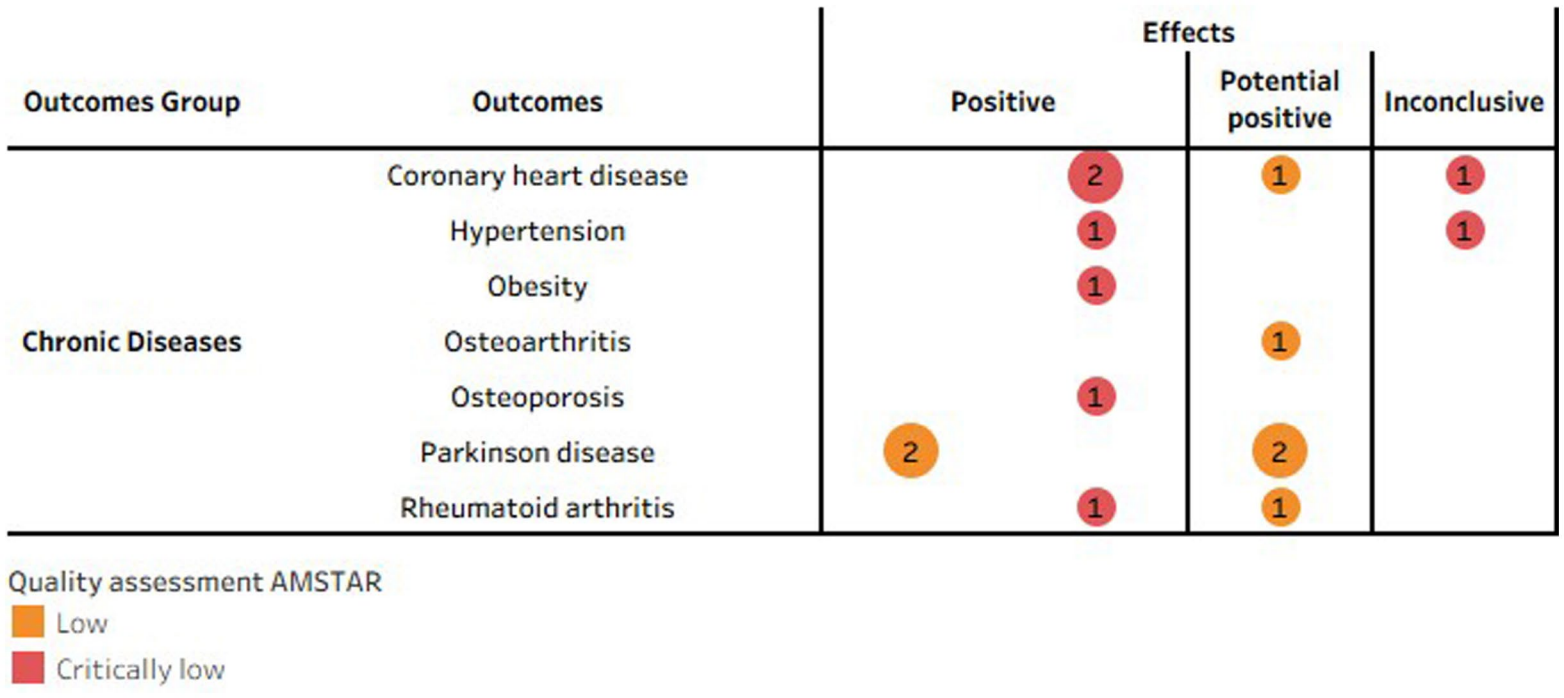
Bubble plot mapping of effectiveness and methodological quality matrix of Tai Chi interventions for specific chronic disease outcomes. Note: This breakdown highlights specific clinical conditions (e.g., coronary heart disease, hypertension, Parkinson’s disease), where bubble size reflects study volume and colors represent AMSTAR confidence levels (Orange = Low; Red = Critically low).

Chronic diseases effects were related to 15 outcomes (Figure 7) related to Parkinson’s disease (4), coronary heart disease (4), hypertension (2), rheumatoid arthritis (2), osteoporosis (1), osteoarthritis (1), and obesity (1). Positive effects were found on Parkinson’s disease (2), coronary heart disease (2), hypertension (1), obesity (1), osteoporosis (1), and rheumatoid arthritis (1). Potential positive effects were related to Parkinson’s disease (2), coronary heart disease (1), rheumatoid arthritis (1), and osteoarthritis (1). Inconclusive effects were shown for hypertension (1) and coronary heart disease (1).

#### 3.6.5 Metabolic indicators

**Figure 8:**
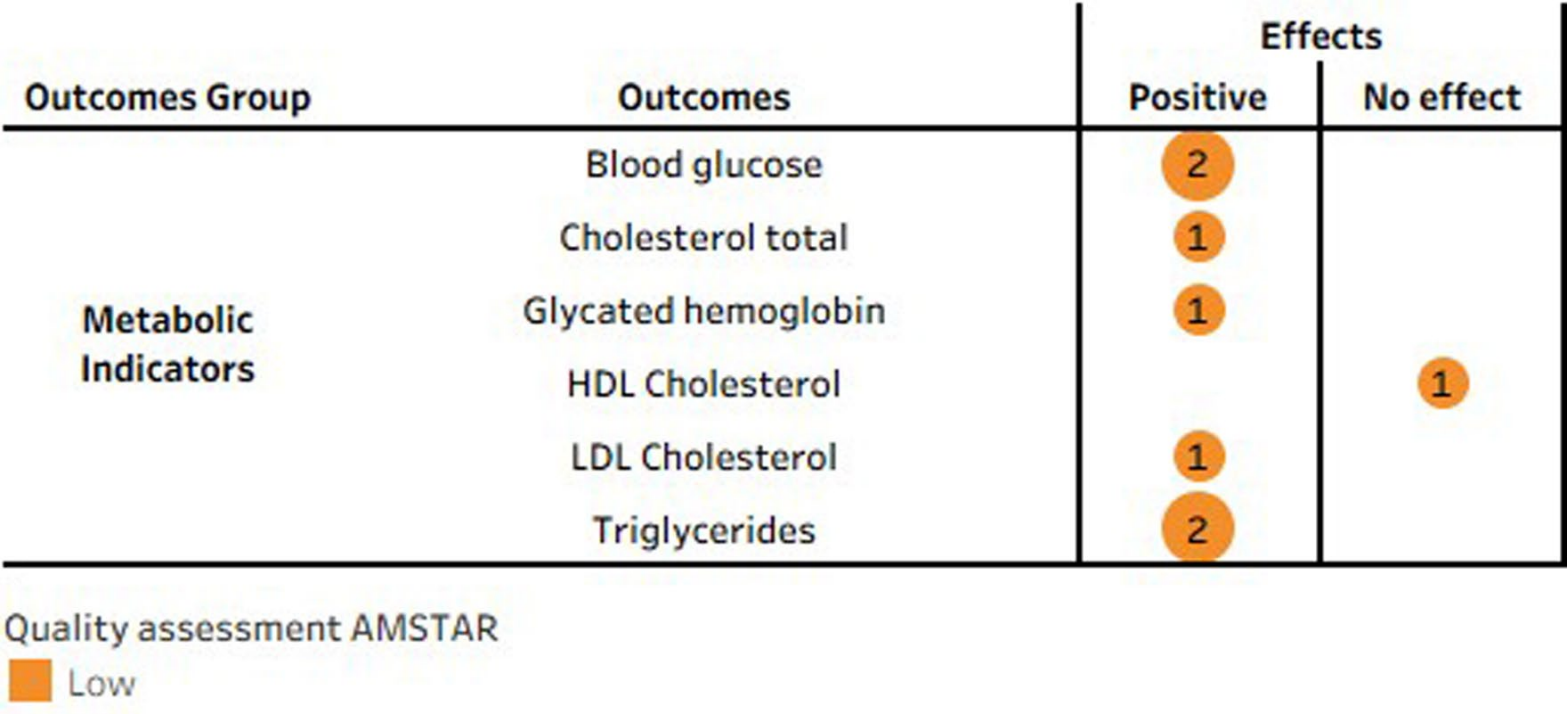
Bubble plot mapping of effectiveness and methodological quality matrix of Tai Chi interventions for specific metabolic indicator outcomes. Note: This breakdown highlights specific metabolic and biochemical metrics (e.g., blood glucose, lipid profile), where bubble size reflects study volume and the color represents the AMSTAR confidence level (Orange = Low).

The metabolic indicators group had eight (08) outcomes (Figure 8), which were related to: blood glucose (2), triglycerides (2), glycated hemoglobin (1), total cholesterol (1), LDL cholesterol (1), and HDL cholesterol (1). Positive effects were found for blood glucose (2), triglycerides (2), total cholesterol (1), glycated hemoglobin (1), and LDL cholesterol (1). We found one HDL cholesterol outcome, where Tai Chi has no effect.

### 3.7 Intervention

**Figure 9:**
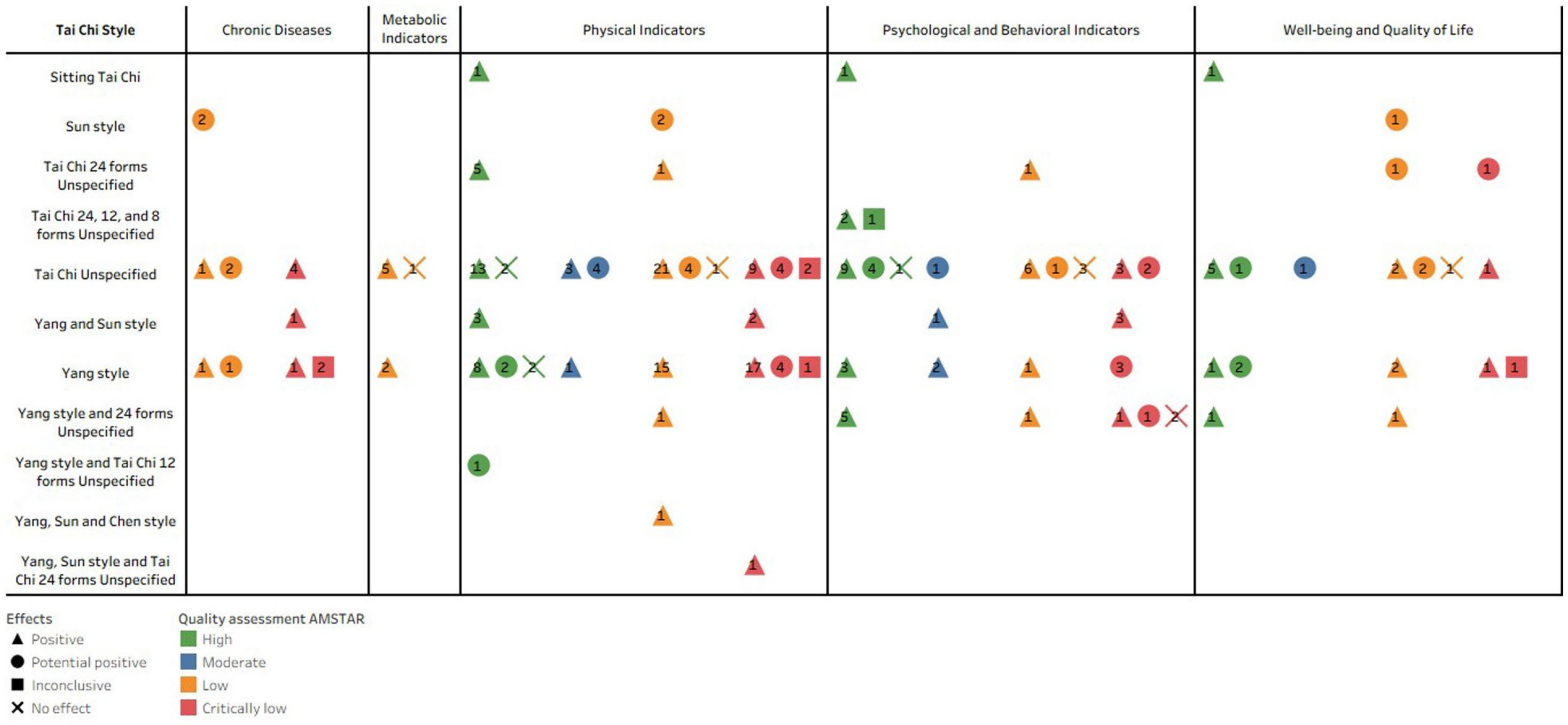
Matrix representation of the most widely used Tai Chi styles, categorized by outcome groups, intervention effects, and methodological quality.

The most widely used style of Tai Chi was the Yang style, two to three times per week, with each session lasting 30 to 60 min for 12 to 24 weeks. However, Chen and Sun styles have also been found, along with various simplified forms. We identify that the effectiveness of Tai Chi may be influenced by specific exercise parameters such as style, duration, and frequency, with parameters sometimes varying depending on the desired health outcome. This Evidence Map can identify the most studied parameters for some common health outcomes in older adults (Figure 9).

### 3.8 Measurement instruments

Several measurement instruments have been commonly used in research to assess a wide range of functions and symptoms in older adults. We have mapped the most commonly used ones, and they are presented by health conditions that they assess below.

#### 3.8.1 Global cognitive function

The Mini-Mental State Examination (MMSE) and Montreal Cognitive Assessment (MoCA) were the most frequently used scales for evaluating global cognitive function, including mild cognitive impairment (MCI) and dementia. Other tools include the Alzheimer’s Disease Assessment Scale-Cognitive subscale (ADAS-Cog) and the Clinical Dementia Rating (CDR).

#### 3.8.2 Balance, physical function, and mobility

The clinical tests Timed Up and Go (TUG), Berg Balance Scale (BBS), Functional Reach Test (FRT), and Single-Leg Stance (SLS) (eyes open/closed) were the most common outcome measures to assess balance, agility, and functional mobility. Other balance and mobility measures include the 6 Minute Walk Test (6MWT), 50-ft walk, Tinetti Performance Oriented Mobility Assessment (POMA), Sit-To-Stand Test, Tandem Stance Test, and Short Physical Performance Battery (SPPB).

#### 3.8.3 Falls and fear of falling

The Falls Efficacy Scale (FES) and Activities-specific Balance Confidence (ABC) Scale were commonly used to measure fear of falling and determine capabilities in performing activities of daily living without falling. Nonetheless, the incidence of falls (rate/number of falls) seems to be considered the most direct indicator of effectiveness in falls prevention.

#### 3.8.4 Motor and non-motor function in Parkinson’s disease

The Unified Parkinson’s Disease Rating Scale (UPDRS) was the most widely used scale to assess Parkinsonian symptoms, including motor symptoms (UPDRS Motor Subscale 3/UPDRS III). Another assessment, the Parkinson’s Disease Questionnaire (PDQ-39), was a common tool used to assess quality of life in Parkinson’s, Parkinson’s Disease Sleep Scale-2 (PDSS-2), which was used to assess sleep disorders, and the Non-Motor Symptoms Scale (NMSS) to assess non-motor symptoms in Parkinson’s disease.

#### 3.8.5 Depression and psychological well-being

A common instrument for evaluating symptoms of older adults’ depression was the Geriatric Depression Scale (GDS) as well as the Beck Depression Inventory (BDI). Other measures include the Hamilton Depression Scale (HAMD), the Centre for Epidemiological Studies Depression Scale (CES-D), and the Montgomery–Asberg Depression Rating Scale (MADRS). Furthermore, the Affect Balance Scale (ABS), Profile of Mood States (POMS), and Self-Efficacy Scale were commonly used to measure emotional well-being or mood.

#### 3.8.6 Quality of life

A widely used generic health survey to measure quality of life commonly used was the 36-Item Short Form Survey (SF-36), the shorter version, SF-12, WHO-QOL-BREF, especially the Chinese version. Other measures involved the Quality of Life Enjoyment and Satisfaction Questionnaire (Q-LES-Q) and the Quality of Life (QOL) KOOS, which were used to assess quality of life specific to osteoarthritis individuals.

#### 3.8.7 Pain and osteoarthritis-related outcomes

A common instrument for evaluating symptoms of older adults’ osteoarthritis was the Western Ontario and McMaster Universities Arthritis Index (WOMAC), which was especially used to assess pain, stiffness, and physical function. The Visual Analog Scale (VAS) was used to assess pain, and the Knee Injury and Osteoarthritis Outcome Score (KOOS), which evaluates pain, symptoms, Activities of Daily Living (ADL), sport/recreation, and knee-related quality of life. Other measures included the Oswestry Disability Index (ODI) to assess pain-associated disability.

#### 3.8.8 Executive function and memory

The Trail-Making Test (TMT) A/B/B-A was used to evaluate processing speed, cognitive flexibility, and task switching. The Digit Span (Forward/Backward) was commonly used to assess working memory, and the Stroop Test (Color-Word) was used to assess inhibition and mental speed. Other tools for executive function and memory were the Auditory Verbal Learning Test (AVLT), the Frontal Assessment Battery (FAB), the Hopkins Verbal Learning Test (HVLT), the Rivermead Behavioral Memory Test (RBMT), and the Wechsler Adult Intelligence Scale (WAIS).

#### 3.8.9 Sleep

The Pittsburgh Sleep Quality Index (PSQI) is commonly used to assess the quality of sleep. Moreover, Polysomnography and Accelerometry were used to measure different sleep stages.

#### 3.8.10 Bone mineral density

Dual X-ray Absorptiometry (DXA) was the primary method for diagnosing osteoporosis by assessing Bone Mineral Density (BMD) at the lumbar spine, proximal femur, and distal radius. Other tools were the High-resolution peripheral quantitative computed tomography (HR-pQCT) and three-dimensional peripheral quantitative computed tomography (3D-pQCT).

#### 3.8.11 Other general measures

Activities of Daily Living (ADL) have been commonly used to assess functional status, Body Mass Index (BMI) to assess a measure of body fat, Range of Motion (ROM) to assess joint movement and flexibility, and the Visual Analogical Scale (VAS) to assess pain, fatigue, or discomfort.

### 3.9 Adverse events

Considering 118 systematic reviews, only five (45, 54, 61, 68, 141) reported adverse events from Tai Chi interventions, which were related to cases of dyspnea, muscle pain, fatigue, ankle sprain, back pain, knee pain, pain, and fall. Ninety-seven studies did not report whether or not there was an adverse event. However, sixteen (37, 39, 40, 49, 51, 52, 55, 56, 65, 70, 91, 93, 121, 134, 145, 146) studies related that Tai Chi interventions do not have adverse effects.

### 3.10 Quality assessment

The systematic reviews were analyzed using the AMSTAR 2 tool and presented the following quality assessment: 36 high, 07 moderate, 42 low, and 33 critically low. Supplementary Table 3 summarizes the setting, participants’ total number, age/range, population diversity, the intervention, outcomes of the reviews evaluated with positive effects and high (31) reliability by the AMSTAR2 tool. All information follows as described in the systematic reviews. Confused or incomplete information was described as “not reported”.

## 4 Discussion

### 4.1 Overview of the evidence base

This evidence map synthesizes findings from 118 systematic reviews and meta-analyses assessing the effects of Tai Chi on health outcomes in older adults. Most of the reviews were published in the last five years. The reviews encompassed a broad range of populations, including community-dwelling older adults, individuals with neurological conditions, physical impairment, mental disorders, and chronic diseases. However, many reviews did not specify the settings in which primary studies were conducted, as well as some that included heterogeneous populations, limiting contextual interpretation.

The EGM developed for this study provides a visual synthesis of these findings, enabling users to examine the distribution of evidence across interventions, health outcomes, Tai Chi styles, population groups, and methodological quality. This visualization also highlights areas with substantial research concentration and those with limited or absent evidence.

While previous evidence maps have explored Tai Chi in general populations, this study is, to our knowledge, the first to focus exclusively on older adults. By centering on this demographic, we provide a more granular analysis of outcomes relevant to gerontologic and geriatric health. Furthermore, this map enforces the evidence base compared to earlier broader syntheses, offering a specialized stratification by style and dosage that was previously unavailable for this age group.

### 4.2 Effects and health outcomes

#### 4.2.1 Physical health

Tai Chi interventions were associated with positive effects on physical health, especially regarding balance and mobility, contributing to fall prevention by reducing fall risk and improving motor function, flexibility, proprioception, and muscle strength across 50 reviews (21, 32–34, 36, 38, 40, 44, 46, 47, 49, 50, 54, 56, 60, 61, 64, 65, 71, 75, 76, 79–82, 85, 88, 90, 97, 99, 100, 105, 108, 110, 113, 116–118, 120–122, 125, 126, 132, 137, 139–141, 143). These findings support its potential role in functional maintenance and fall prevention among older adults. Benefits with positive effects were also reported in individuals with chronic conditions, including rheumatoid arthritis, osteoporosis, chronic pain (joint and osteoporotic pain), coronary heart disease, hypertension, obesity, and osteopenia (35, 37, 48, 57, 61, 65, 69, 91, 92, 94, 113, 131, 134). Other positive effects, such as exercise capacity, blood pressure, and heart rate, were also found, but with insignificant representation (21, 34, 45, 57, 72, 92, 94, 114). Inconclusive effects were reported related to balance in one systematic review that evaluated just the instructional Tai Chi method (62). No effects were observed regarding muscle mass in sarcopenic older adults (142), nor were improvements noted in the agility and mobility of older individuals with Parkinson’s disease (40, 85). Regarding the improvement of muscle mass in older adults with sarcopenia, there is stronger evidence supporting resistance training and its combination with protein intake (148–151); conversely, for mobility and functional ability in Parkinson’s disease, repetitive transcranial magnetic stimulation (rTMS) combined with rehabilitation therapies demonstrate superior evidence (152–155).

#### 4.2.2 Psychological and behavioral indicators

Reviews reported positive effects on psychological outcomes, including reductions in depressive symptoms, anxiety, fear of falling, and negative emotions (21, 82, 96, 101, 104, 109, 114, 119, 123, 138). Furthermore, positive effects were observed in neurological parameters, specifically regarding motor functions in Parkinson’s disease, global cognitive function in individuals with and without dementia, executive function, visuospatial ability, attention, and memory performance (70, 73, 84, 89, 95, 98, 99, 103, 106, 107, 112, 124, 128–130, 139, 145, 146). Given the rising global prevalence of cognitive impairment, Tai Chi emerges as a strategic tool for the management of dementia processes. By improving executive function, attention, and memory, this intervention can mitigate the burden of neurodegenerative diseases on public health systems and improve the quality of life for an aging population facing an increasing frequency of dementia diagnoses (156). Inconclusive effects were reported related to the prevention of stroke and memory (58, 102), and no effects were related to concentration, memory, depression, and global cognitive function (104, 115, 128–130). However, these findings contrast with the positive effects reported in most other reviews. This discrepancy may be attributed to high population heterogeneity in the primary studies, which often included participants with diverse clinical profiles and varying health statuses. Nevertheless, for stroke prevention, evidence is more established for comprehensive lifestyle modifications. These include regular moderate-to-vigorous physical activity, adherence to the Mediterranean diet, and smoking cessation, alongside pharmacological management for blood pressure control and diabetes (157–159).

#### 4.2.3 Well-being and quality of life

Beyond physical improvements, Tai Chi offers significant well-being and quality of life benefits, including enhanced sleep quality, improved activities of daily living, and overall patient safety and quality of life (44, 47, 74, 86, 87). Notably, the practice also holds potential for enhancing socialization among older adults regarding its impact on social interaction, improving social connectivity, and remains a factor in promoting psychological well-being and reducing isolation in this population. The potential of Tai Chi for socialization is attributed to its nature as a group-based intervention contributing to a more holistic approach to healthy aging (77).

#### 4.2.4 Other conditions

Regarding other conditions, Tai Chi has demonstrated positive effects on metabolic markers, including blood glucose, triglycerides, glycated hemoglobin, total cholesterol, and LDL cholesterol. However, no effects on HDL cholesterol were found. It is important to note that these metabolic outcomes were poorly represented in the reviews, which limits the strength of the evidence for these specific findings.

### 4.3 Tai Chi styles and outcomes

The Yang style was the most frequently studied modality, demonstrating consistent benefits for physical, mental, and neurological indicators, and well-being. Its wide older adult clinical use is attributed to its gentle and continuous nature. In contrast, evidence for the Chen and Sun styles remains limited and heterogeneous. The Sun style is characterized by higher stances and “follow-step” footwork, while the Chen style involves more explosive power. The frequent omission of specific details regarding style and intensity in the literature prevents the establishment of definitive dose–response relationships.

Furthermore, only one systematic review included a sitting Tai Chi protocol (114), with 630 older adult women, which indicated potential benefits for upper-limb function and balance in populations with mobility limitations. While this evidence remains preliminary, sitting Tai Chi protocols represent a promising and feasible intervention for nursing home residents, who often present significant functional limitations and could benefit from adapted mind–body practices. Nevertheless, further high-quality studies are needed to consolidate these findings in this specific setting.

### 4.4 Intervention parameters

Intervention parameters such as Tai Chi style, session frequency, duration, and program length appear to modulate outcomes. The Yang style, two to three times per week, with each session lasting 30 to 60 min for 12 to 24 weeks, was the most commonly studied format. Chen and Sun’s styles were also utilized, indicating the need for intervention standardization according to specific clinical positive effects goals such as rheumatoid arthritis, global cognitive function, muscle strength, negative emotions, risk of fall, and chronic pain (61, 70, 80, 104, 108, 126, 134).

### 4.5 Tai Chi and intrinsic capacity in older adults

The evidence synthesized in this map indicates that Tai Chi interventions have the potential to improve domains of Intrinsic Capacity (IC), although effects vary by Tai Chi style, population characteristics, and intervention parameters. This EGM shows that most of the available research is concentrated in the domains of mobility, vitality, and cognition, with far fewer studies addressing psychological well-being and sensory function.

Considering mobility and physical function, systematic reviews and meta-analyses show benefits of Tai Chi on balance, gait, overall physical function, and muscle strength in older adults (81, 82, 110, 127). A 2024 meta-analysis demonstrated significant improvements in Timed Up and Go (TUG), Functional Reach, and Berg Balance Scale scores among healthy older adults, especially when Tai Chi was practiced ≥2 times per week in ≥45-min sessions (132). In comparing different modalities, Yang style Tai Chi demonstrated superior and more consistent effects on mobility, balance, and general physical outcomes when compared to Chen, Sun, and other simplified forms (140, 141).

The evidence also supports cognitive benefits. Systematic reviews reported improvements in executive function, memory, visuospatial ability, and global cognition among older adults with mild cognitive impairment following Tai Chi training (70, 84, 89, 95, 98, 103, 106, 107, 112, 124, 128–130, 135, 145, 146).

Together, these results suggest that Tai Chi may promote or preserve multiple domains of IC (mobility, physical fitness, cognitive performance, and vitality), particularly when practiced in styles and doses aligned with functional goals.

### 4.6 Quality assessment considerations

Methodological quality varied substantially, and only 31 systematic reviews achieved high quality on AMSTAR 2. Common limitations included insufficient reporting (e.g., population details, setting) and heterogeneity in intervention protocols. This EGM integrates quality assessments directly into its visualization, allowing users to identify areas supported by high confidence evidence and areas where findings rely on low or critically low quality reviews. It is worth noting that reviews with low or critically low methodological quality were predominantly older publications.

### 4.7 Evidence gaps

#### 4.7.1 Population gaps

Population gaps were that adults aged 80 years and older were rarely examined, despite representing a growing demographic with distinct functional and clinical needs. Individuals receiving palliative care were almost absent from the literature, although Tai Chi may offer supportive benefits for symptom management and well-being. Rehabilitation-focused populations (e.g., post-stroke, post-fracture, post-surgical patients) were minimally represented, limiting conclusions about Tai Chi as a rehabilitation modality. Finally, there is a notable lack of representation for populations in rural settings.

#### 4.7.2 Gender gaps

This EGM demonstrated that the effect of Tai Chi on health outcomes may be gender-dependent; however, the generalization of these results should be approached with caution. Our findings reveal a significant reporting gap, as 63 reviews did not clarify the gender distribution of their participants. Although 42 reviews identified a female majority, the underrepresentation of male-dominant samples (7) and the lack of sex-disaggregated data in 63 reviews hinder a nuanced understanding of how Tai Chi affects men and women.

#### 4.7.3 Intervention gaps

Although this EGM demonstrates that certain Tai Chi styles are linked to specific health outcomes, limited direct comparisons between styles preclude identifying style-specific patterns for particular clinical conditions. Seated Tai Chi remains underexplored, despite its relevance for mobility-restricted or frail populations. Data on dose–response relationships and long-term effects remain insufficient due to inconsistent reporting of frequency, intensity, and program duration.

A notable Brazilian regional intervention gap was observed regarding the Tai Chi Pai Lin style. Although it is widely integrated into public health policies in Brazil, it remains virtually absent from international indexed databases. The term is currently unrecognized by major repositories such as PubMed, and our search indicated a total lack of systematic reviews on this modality. This absence prevents the inclusion of Pai Lin in global evidence syntheses and highlights a significant disconnect between widespread community practice in South America and its formal scientific validation.

Preliminary evidence from regional literature and institutional reports on Tai Chi Pai Lin indicates a significant impact on health promotion within the Brazilian Unified Health System (SUS) (160). Studies focusing on public health users suggest that the practice is associated with improved functional fitness and quality of life among older adults, as well as a perceived reduction in medication use for chronic conditions. The modality is reported as a resource for mental health, social interaction, and self-care, particularly in primary care settings. Among the older adults, the practice is often mediated by a philosophical framework of “peace and healing,” contributing to active aging and the humanization of healthcare (161–165). However, these findings are primarily documented in descriptive studies, qualitative meta-syntheses, and narrative reviews, underscoring the lack of high-level quantitative evidence for international scientific standardization.

#### 4.7.4 Outcome gaps

This EGM identified research gaps regarding cardiometabolic outcomes (HDL cholesterol and stroke prevention), muscle mass and strength in older adults with sarcopenia and frailty, and agility and mobility in Parkinson’s disease. Specific cognitive and mental health gaps were noted for concentration and memory in individuals with mild cognitive impairment. Notably, depression outcomes (2) lacked evidence in older adults not using medication (Tai Chi as monotherapy) and in those with dementia. Furthermore, a shortage of long-term follow-up data remains across these clinical areas. Finally, a methodological limitation observed was the conflation of healthy older adults with those presenting some specific health conditions. This lack of stratification by clinical status can make it difficult to determine the intervention’s effects for this specific population. Additionally, adverse event reporting remained infrequent across the analyzed reviews.

#### 4.7.5 Instructor qualifications

The systematic reviews included in this evidence map provided limited information on the professional training or technical qualifications of Tai Chi instructors. This gap precludes assessment of instructor standardization, certification levels, or experience as potential effect modifiers across interventions. Future primary studies should report instructor credentials to enable evaluation of practitioner expertise as a source of intervention heterogeneity.

### 4.8 Implications for research and practice

Future research should prioritize large, well-designed randomized controlled trials directly comparing Tai Chi styles and adhering to standardized reporting guidelines. Studies focusing on populations aged 80+, rural populations, palliative care settings, and rehabilitation contexts would address critical evidence gaps identified through the EGM. From a public health perspective, Tai Chi remains a low-cost, sustainable, and accessible practice with potential to support functional, psychological, and chronic disease-related outcomes in aging populations (166–170).

Furthermore, there is a need to formalize the study of regional Tai Chi modalities in Brazil, such as the Pai Lin style, to overcome current indexing barriers in databases like PubMed. Given its high adherence in primary care settings, researchers should prioritize high-quality clinical trials that utilize standardized terminology to ensure international visibility. Validating such regional styles is essential for establishing culturally adapted protocols and ensuring that traditional knowledge is supported by rigorous, evidence-based frameworks within public health systems.

### 4.9 Limitations and strengths

The findings are recommended with caution due to limitations in methodology, related to heterogeneity among studies, and small sample sizes. Furthermore, the level of expertise of Tai Chi instructors and the standardization of Tai Chi protocols (style, dose, teaching methods) can influence outcomes. Clear descriptions of the intervention are often lacking. While optimal parameters are suggested, individuals, especially older adults with chronic conditions, should select an exercise program that aligns with their interests, preferences, and physical condition, ideally under professional guidance.

In addition, the grouping of outcomes was review-content driven. Even though individual primary research studies would have more contributions to add to the analysis, this was not the focus of the Map. Besides, we were unable to avoid overlapping the included studies across reviews, but we did not repeat results from updated reviews. We relied on the review author’s skills in conducting systematic reviews, evaluation of primary studies’ quality, choice of outcomes, analysis of effects and susceptibility to publication, and outcome reporting bias. To mitigate this dependency and enhance the reliability of our conclusions, we applied the AMSTAR 2 tool to critically appraise the confidence of the included systematic reviews.

We acknowledge the importance of the Corrected Covered Area (CCA) in *Overviews of Reviews*, particularly when the goal is to conduct a meta-analysis of second-order effects. However, this EGM was designed as an Evidence and Gap Map (EGM) following the Campbell Collaboration guidelines (24).

According to the Campbell methodology, the primary purpose of an EGM is to provide a visual representation of the evidence landscape and identify research “gaps” and “gluts” to inform policy and future research agendas. As our synthesis is strictly qualitative and focused on the distribution of systematic reviews across interventions and outcomes, the calculation of the CCA was not deemed mandatory for achieving the map’s diagnostic objectives.

This study has certain limitations inherent to the EGM methodology. Following the Campbell Collaboration framework, this map aims to identify the availability of evidence rather than to produce a new pooled effect size. Consequently, we did not calculate the Corrected Covered Area (CCA). While it is likely that several primary clinical trials are included in multiple systematic reviews, particularly in well-studied areas of Tai Chi for falls prevention, balance, and global cognitive function, this redundancy does not compromise the identification of research gaps or the mapping of the evidence architecture. However, readers should interpret the ‘density’ of evidence in certain nodes of the map with caution, as it may reflect a high frequency of reviews rather than a large volume of unique primary studies. Future research focusing on a quantitative synthesis of these reviews should consider applying the CCA to adjust for such overlaps.

Evidence maps are not designed to provide detailed and definitive information on the effectiveness of interventions. The implementation of the reviewed interventions in practice will require additional steps (e.g., identifying the optimal intervention format). Generally, evidence maps are a very broad overview of the evidence base, indicating areas in which research has been conducted, to help stakeholders interpret the state of the evidence to inform policy and clinical decision-making.

This EGM can provide a broad research overview of Tai Chi to older adults; the findings showed more positive effects and none potential negative or negative ones.

The creation and publication of this EGM consists of graphically representing the best evidence found, analyzed, and categorized, in addition to linking with the bibliographic records and full texts (when available) of the studies in order to facilitate access to information for all those interested.

## 5 Conclusion

In summary, Tai Chi demonstrates robust positive effects on physical conditions, particularly regarding balance, motor function, muscle strength, and fall risk. Significant benefits were also identified for mental and neurological health, including Parkinson’s disease, depression, global cognitive function, well-being, and sleep quality. While common protocols range from 30 to 60 min per session (3 to 5 times per week for 12 to 24 weeks), specific outcomes such as metabolic health and frailty may require higher frequencies.

**Figure 10:**
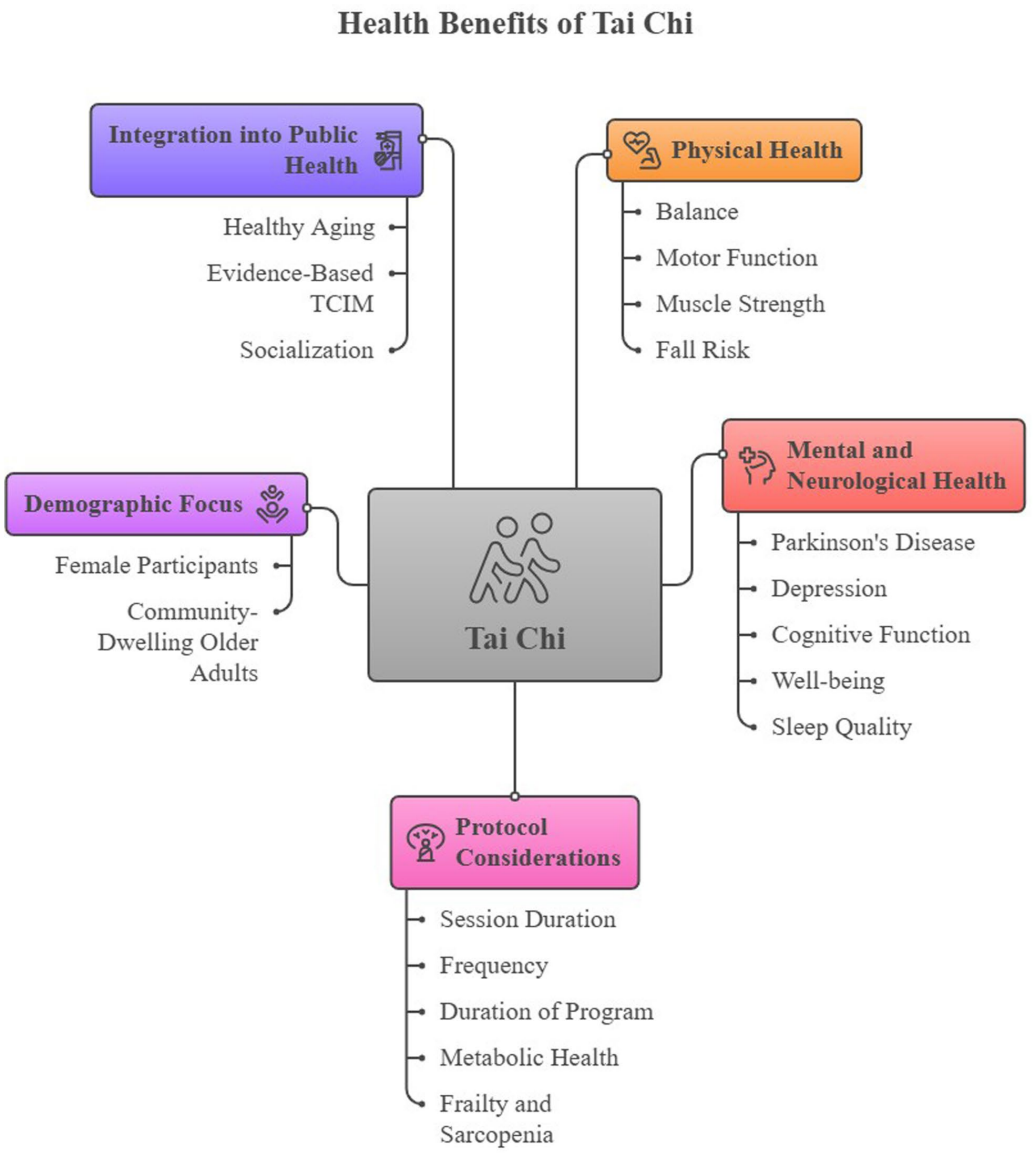
Synthesis diagram of Tai Chi health dimensions, target demographics, and research gaps. Note: This conceptual map integrates physical, mental, and public health outcomes with critical protocol considerations (e.g., dosage, duration) to guide future evidence-based interventions.

The Yang style remains the most frequently studied modality. The current body of evidence is based especially on female participants and community-dwelling older adults, a demographic concentration that must be considered when designing and implementing new protocols (Figure 10). Given these findings, Tai Chi should be considered for integration into public health services to promote healthy aging and socialization through evidence-based Traditional, Complementary, and Integrative Medicine (TCIM). This map identifies 171 positive and promising health outcomes. It serves as a foundational tool for patients, practitioners, and policymakers to implement therapies tailored to the specific needs of the aging population.
