# Supplementary material for: Evidence map of Tai Chi interventions for older adults
Source: Front Public Health. 2026 Jul 2;14:1820294. doi: 10.3389/fpubh.2026.1820294 (PMC13372770; doi:10.3389/fpubh.2026.1820294)
Supplement: Supplementary file 3 [file Table_1.docx]

| **Database** | **Tai Chi** |
| --- | --- |
| **1 PUBMED** | tai chi chuan [tiab] OR taijiquan OR taiji OR ai chi [tiab] OR tai chi [tiab] OR tai ji [tiab] OR tai chi quan [tiab] OR tai chi [tiab] OR taijiquan [tiab] |
|  | **Older Adult** |
|  | "aged"[Title/Abstract] OR "aged"[MeSH Terms] OR "geriatric*"[Title/Abstract] OR "geriatrics"[MeSH Terms] OR "elder*"[Title/Abstract] OR senior citizen [tiab] OR old age person [tiab] OR old age pensioner [tiab] OR oldest [tiab] OR geriatric [tiab] OR Elderly [tiab] OR homes for the aged [mh] OR aged, 80 and over [mh] OR Elderly [tiab] OR community-dwelling [tiab] OR geriatric [tiab] OR “mini-mental state” [tiab] OR alzheimer [tiab] OR alzheimer’s [tiab] OR alzheimers [tiab] OR mmse [tiab] OR Frailty [tiab] OR Gds [tiab] OR Ageing [tiab] OR “hip fractures “[tiab] OR elders [tiab] OR Frail [tiab] OR Demented [tiab] OR Psychogeriatrics [tiab] OR gerontolog* [tiab] OR “cognitive impairment” [tiab] OR “postmenopausal women” [tiab] OR comorbidities [tiab] OR dementia [tiab] OR aging [tiab] OR older [tiab] OR “daily living” [tiab] OR “cognitive decline” [tiab] OR “cognitive impairment” [tiab] OR “cognitive functioning” OR “old people” [tiab] OR nursing homes [mh] OR Geriatric assessment [mh] OR aging [mh] OR frail elderly [mh] OR Alzheimer disease [mh] OR homes for the aged [mh] OR cognition disorders [mh] OR dementia [mh] OR Activities of daily living [mh] OR ageing[TIAB] OR aging[TIAB] OR aging[MESH] OR "frail elderly"[MESH] |
|  | **Review** |
|  | ("Systematic Review"[Publication Type:NoExp] OR "Systematic Reviews as Topic"[mesh:noexp] OR (("comprehensive"[TIAB] OR "integrated"[TIAB] OR "integrative"[TIAB] OR "mapping"[TIAB] OR "methodology"[TIAB] OR "narrative"[TIAB] OR "scoping"[TIAB] OR "systematic"[TIAB]) AND ("search"[TIAB] OR "searched"[TIAB] OR "searches"[TIAB] OR "studies"[TIAB]) AND ("cinahl"[TIAB] OR "cochrane"[TIAB] OR "embase"[TIAB] OR "psycinfo"[TIAB] OR "pubmed"[TIAB] OR "medline"[TIAB] OR "scopus"[TIAB] OR "web science"[TIAB] OR "bibliographic review"[TIAB:~1] OR "bibliographic reviews"[TIAB:~1] OR "literature review"[TIAB:~1] OR "literature reviews"[TIAB:~1] OR "literature search"[TIAB:~1] OR "literature searches"[TIAB:~1] OR "narrative review"[TIAB:~1] OR "narrative reviews"[TIAB:~1] OR "qualitative review"[TIAB:~1] OR "qualitative reviews"[TIAB:~1] OR "quantitative review"[TIAB] OR "quantitative reviews"[TIAB])) OR "comprehensive review"[TIAB] OR "comprehensive reviews"[TIAB] OR "comprehensive search"[TIAB] OR "comprehensive searches"[TIAB] OR "critical review"[TIAB] OR "critical reviews" [TIAB] OR (("electronic database"[TIAB:~1] OR "electronic databases"[TIAB:~1] OR "databases searched"[TIAB:~3]) AND (eligibility[tiab] OR excluded[tiab] OR exclusion[tiab] OR included[tiab] OR inclusion[tiab])) OR "evidence assessment"[TIAB] OR "evidence review"[TIAB] OR "exploratory review"[TIAB] OR "framework synthesis"[TIAB] OR "Integrated review"[TIAB] OR "integrated reviews"[TIAB] OR "integrative review"[TIAB:~1] OR "integrative reviews"[TIAB:~1] OR "mapping review"[TIAB:~1] OR "meta-review"[TIAB:~1] OR "meta-synthesis"[TIAB:~1] OR "methodology review"[TIAB:~1] OR ("mixed methods"[TIAB:~0] AND "methods review"[TIAB:~1]) OR ("mixed methods"[TIAB:~0] AND "methods synthesis"[TIAB:~1]) OR "overview reviews"[TIAB:~4] OR ("PRISMA"[TIAB] AND "preferred"[TIAB]) OR "PRISMA-P"[TIAB:~0] OR "prognostic review"[TIAB:~1] OR "psychometric review"[TIAB:~1] OR ("rapid evidence"[TIAB:~0] AND "evidence assessment"[TIAB:~0]) OR "rapid realist"[TIAB:~0] OR "rapid review"[TIAB:~1] OR "rapid reviews"[TIAB:~1] OR "realist review"[TIAB:~1] OR "review of reviews"[TIAB:~1] OR "scoping review"[TIAB:~1] OR "scoping reviews"[TIAB:~1] OR "scoping study"[TIAB:~1] OR ("state art "[TIAB:~2] AND "art review"[TIAB:~1]) OR "systematic evidence map"[TIAB] OR "systematic mapping"[TIAB:~1] OR "systematic literature"[TIAB:~1] OR "systematic Medline"[TIAB:~2] OR "systematic PubMed"[TIAB:~2] OR "systematic review"[TIAB:~1] OR "systematic reviews"[TIAB:~1] OR "systematic search"[TIAB:~1] OR "systematic searches"[TIAB:~1] OR "systematical review"[TIAB:~1] OR "systematical reviews"[TIAB:~1] OR "systematically identified"[TIAB:~1] OR "systematically review"[TIAB:~1] OR "systematically reviewed"[TIAB:~1] OR "umbrella review"[TIAB:~1] OR "umbrella reviews"[TIAB:~1] OR "Cochrane Database Syst Rev"[ta] OR "evid rep technol assess full rep"[Journal] OR "evid rep technol assess summ"[Journal]) |
| **Database** | **Tai Chi** |
| **2 EMBASE** | 'tai chi chuan':ti,ab,kw OR 'taijiquan' OR 'taiji' OR 'ai chi':ti,ab,kw OR 'tai chi':ti,ab,kw OR 'tai ji':ti,ab,kw OR 'tai chi quan':ti,ab,kw OR 'tai chi':ti,ab,kw OR 'taijiquan':ti,ab,kw |
|  | **Older adult** |
|  | 'aged':ti,ab,kw OR 'aged'/exp OR 'geriatric*':ti,ab,kw OR 'geriatrics'/exp OR 'elder*':ti,ab,kw OR 'senior citizen':ti,ab,kw OR 'old age person':ti,ab,kw OR 'old age pensioner':ti,ab,kw OR 'oldest':ti,ab,kw OR 'geriatric':ti,ab,kw OR 'elderly':ti,ab,kw OR 'home for the aged'/exp OR 'senescence'/exp OR 'elderly':ti,ab,kw OR 'community-dwelling':ti,ab,kw OR 'geriatric':ti,ab,kw OR 'mini-mental state':ti,ab,kw OR 'alzheimer':ti,ab,kw OR 'alzheimers':ti,ab,kw OR 'mmse':ti,ab,kw OR 'frailty':ti,ab,kw OR 'gds':ti,ab,kw OR 'ageing':ti,ab,kw OR 'hip fractures':ti,ab,kw OR 'elders':ti,ab,kw OR 'frail':ti,ab,kw OR 'demented':ti,ab,kw OR 'psychogeriatrics':ti,ab,kw OR 'gerontolog*':ti,ab,kw OR 'cognitive impairment':ti,ab,kw OR 'postmenopausal women':ti,ab,kw OR 'comorbidities':ti,ab,kw OR 'dementia':ti,ab,kw OR 'aging':ti,ab,kw OR 'older':ti,ab,kw OR 'daily living':ti,ab,kw OR 'cognitive decline':ti,ab,kw OR 'cognitive impairment':ti,ab,kw OR 'cognitive functioning' OR 'old people':ti,ab,kw OR 'nursing home'/exp OR 'geriatric assessment'/exp OR 'aging'/exp OR 'frail elderly'/exp OR 'Alzheimer disease'/exp OR 'home for the aged'/exp OR 'cognitive defect'/exp OR 'dementia'/exp OR 'daily life activity'/exp OR 'ageing':ti,ab,kw OR 'aging':ti,ab,kw OR 'aging'/exp OR 'frail elderly'/exp |
| **Database** | **Tai Chi** |
| **3 CINAHL** | AB tai chi chuan OR taijiquan OR taiji OR AB ai chi OR TI tai chi OR AB tai ji OR AB tai chi quan OR AB tai chi AB taijiquan |
|  | **Older adult** |
|  | TI ( (gerontolog*) OR AB (gerontolog*) OR SU (gerontolog*) ) OR TX ( sexagenarian or septuagenarian or octogenarian or nonagenarian or centenarian or (Age# n3 (over or older) n2 (50 or 55 or 60 or 65 or 70 or 75 or 80 or 85 or 90 or 95)) ) OR MH Geriatrics OR TI ( (older n1 (adult# or m?n or wom?n or person# or people)) OR AB (older n1 (adult# or m?n or wom?n or person# or people)) OR SU (older n1 (adult# or m?n or wom?n or person# or people)) ) OR TI ( (elderly or geriatric* or senior) OR AB (elderly or geriatric* or senior) OR SU (elderly or geriatric* or senior) ) OR MH Aged+ |
|  | **Review** |
|  | (MH "meta analysis" OR MH "systematic review" OR MH "Technology, Medical/EV" OR PT "systematic review" OR PT "meta analysis" OR (((TI systematic* OR AB systematic*) N3 ((TI review* OR AB review*) OR (TI overview* OR AB overview*))) OR ((TI methodologic* OR AB methodologic*) N3 ((TI review* OR AB review*) OR (TI overview* OR AB overview*)))) OR (((TI quantitative OR AB quantitative) N3 ((TI review* OR AB review*) OR (TI overview* OR AB overview*) OR (TI synthes* OR AB synthes*))) OR ((TI research OR AB research) N3 ((TI integrati* OR AB integrati*) OR (TI overview* OR AB overview*)))) OR (((TI integrative OR AB integrative) N3 ((TI review* OR AB review*) OR (TI overview* OR AB overview*))) OR ((TI collaborative OR AB collaborative) N3 ((TI review* OR AB review*) OR (TI overview* OR AB overview*))) OR ((TI pool* OR AB pool*) N3 (TI analy* OR AB analy*))) OR ((TI "data synthes*" OR AB "data synthes*") OR (TI "data extraction*" OR AB "data extraction*") OR (TI "data abstraction*" OR AB "data abstraction*")) OR ((TI handsearch* OR AB handsearch*) OR (TI "hand search*" OR AB "hand search*")) OR ((TI "mantel haenszel" OR AB "mantel haenszel") OR (TI peto OR AB peto) OR (TI "der simonian" OR AB "der simonian") OR (TI dersimonian OR AB dersimonian) OR (TI "fixed effect*" OR AB "fixed effect*") OR (TI "latin square*" OR AB "latin square*")) OR ((TI "met analy*" OR AB "met analy*") OR (TI metanaly* OR AB metanaly*) OR (TI "technology assessment*" OR AB "technology assessment*") OR (TI HTA OR AB HTA) OR (TI HTAs OR AB HTAs) OR (TI "technology overview*" OR AB "technology overview*") OR (TI "technology appraisal*" OR AB "technology appraisal*")) OR ((TI "meta regression*" OR AB "meta regression*") OR (TI metaregression* OR AB metaregression*)) OR (TI meta-analy* OR TI metaanaly* OR TI "systematic review*" OR TI "biomedical technology assessment*" OR TI "bio-medical technology assessment*" OR AB meta-analy* OR AB metaanaly* OR AB "systematic review*" OR AB "biomedical technology assessment*" OR AB "bio-medical technology assessment*" OR MW meta-analy* OR MW metaanaly* OR MW "systematic review*" OR MW "biomedical technology assessment*" OR MW "bio-medical technology assessment*") OR ((TI medline OR AB medline OR MW medline) OR (TI cochrane OR AB cochrane OR MW cochrane) OR (TI pubmed OR AB pubmed OR MW pubmed) OR (TI medlars OR AB medlars OR MW medlars) OR (TI embase OR AB embase OR MW embase) OR (TI cinahl OR AB cinahl OR MW cinahl)) OR (SO Cochrane OR SO health technology assessment OR SO evidence report) OR ((TI comparative OR AB comparative) N3 ((TI efficacy OR AB efficacy) OR (TI effectiveness OR AB effectiveness))) OR ((TI "outcomes research" OR AB "outcomes research") OR (TI "relative effectiveness" OR AB "relative effectiveness")) OR (((TI indirect OR AB indirect) OR (TI "indirect treatment" OR AB "indirect treatment") OR (TI mixed-treatment OR AB mixed-treatment) OR (TI bayesian OR AB bayesian)) N3 (TI comparison* OR AB comparison*)) OR ((TI multi* OR AB multi*) N3 (TI treatment OR AB treatment) N3 (TI comparison* OR AB comparison*)) OR ((TI mixed OR AB mixed) N3 (TI treatment OR AB treatment) N3 ((TI meta-analy* OR AB meta-analy*) OR (TI metaanaly* OR AB metaanaly*))) OR (TI "umbrella review*" OR AB "umbrella review*") OR ((TI multi* OR AB multi*) N2 (TI paramet* OR AB paramet*) N2 (TI evidence OR AB evidence) N2 (TI synthesis OR AB synthesis)) OR ((TI multiparamet* OR AB multiparamet*) N2 (TI evidence OR AB evidence) N2 (TI synthesis OR AB synthesis)) OR ((TI multi-paramet* OR AB multi-paramet*) N2 (TI evidence OR AB evidence) N2 (TI synthesis OR AB synthesis)) |
| **Database** | **Tai Chi** |
| **4 SCOPUS** | "tai chi chuan" OR taijiquan OR taiji OR "ai chi" OR "tai chi" OR "tai ji" OR "tai chi quan" OR "tai chi" OR taijiquan |
|  | **Older adult** |
|  | elderly OR oldest OR aging OR aged |
|  | **Review** |
|  | TITLE-ABS-KEY((systematic* W/3 (review* OR overview* )) OR (methodologic* W/3 (review* OR overview* ))) OR TITLE-ABS-KEY((quantitative W/3 (review* OR overview* OR synthes* )) OR (research W/3 (integrati* OR overview* ))) OR TITLE-ABS-KEY((integrative W/3 (review* OR overview* )) OR (collaborative W/3 (review* OR overview* )) OR (pool* W/3 analy* )) OR TITLE-ABS-KEY("data synthes*" OR "data extraction*" OR "data abstraction*" ) OR TITLE-ABS-KEY(handsearch* OR "hand search*" ) OR TITLE-ABS-KEY("mantel haenszel" OR peto OR "der simonian" OR dersimonian OR "fixed effect*" OR "latin square*" ) OR TITLE-ABS-KEY("met analy*" OR metanaly* OR "technology assessment*" OR HTA OR HTAs OR "technology overview*" OR "technology appraisal*" ) OR TITLE-ABS-KEY("meta regression*" OR metaregression* ) OR TITLE-ABS-KEY(meta-analy* OR metaanaly* OR "systematic review*" OR "biomedical technology assessment*" OR "bio-medical technology assessment*" ) OR TITLE-ABS-KEY(medline OR cochrane OR pubmed OR medlars OR embase OR cinahl ) OR SRCTITLE(cochrane OR (health W/2 "technology assessment" ) OR "evidence report" ) OR TITLE-ABS-KEY(comparative W/3 (efficacy OR effectiveness )) OR TITLE-ABS-KEY("outcomes research" OR "relative effectiveness" ) OR TITLE-ABS-KEY((indirect OR "indirect treatment" OR mixed-treatment OR bayesian ) W/3 comparison* ) OR TITLE-ABS-KEY(multi* W/3 treatment W/3 comparison* ) OR TITLE-ABS-KEY(mixed W/3 treatment W/3 (meta-analy* OR metaanaly* )) OR TITLE-ABS-KEY("umbrella review*") OR TITLE-ABS-KEY(multi* W/2 paramet* W/2 evidence W/2 synthesis ) OR TITLE-ABS-KEY(multiparamet* W/2 evidence W/2 synthesis ) OR TITLE-ABS-KEY(multi-paramet* W/2 evidence W/2 synthesis ) |
| **Database** | **Tai Chi** |
| **5 Web of Science** | "tai chi chuan" OR taijiquan OR taiji OR "ai chi" OR "tai chi" OR "tai ji" OR "tai chi quan" OR "tai chi" OR taijiquan |
|  | (Older adult) and Older Adults (Should – Search within topic) and Aging (Should – Search within topic) and Frailty (Should – Search within topic) and Elderly (Should – Search within topic) and Aged (Should – Search within topic) and Older Adult (Should – Search within topic) and Older People (Should – Search within topic) and Falls (Should – Search within topic) and Dementia (Should – Search within topic) and Sarcopenia (Should – Search within topic) and Alzheimer S Disease (Should – Search within topic) and Mild Cognitive Impairment (Should – Search within topic) |
