# Supplementary material for: Evidence map of Tai Chi interventions for older adults
Source: Front Public Health. 2026 Jul 2;14:1820294. doi: 10.3389/fpubh.2026.1820294 (PMC13372770; doi:10.3389/fpubh.2026.1820294)
Supplement: Supplementary file 5 [file Table_3.docx]

Table 3 Setting, participants' total number, age/range, population diversity, intervention, outcomes of the reviews evaluated with positive effects and high reliability by the AMSTAR2.

| REF. | SETTING | TOTAL PARTICIPANTS | AGE RANGE / AGE | POPULATION DESCRIPTION | INTERVENTION DESCRIPTION | OUTCOME | EFFECT | AMSTAR |
| --- | --- | --- | --- | --- | --- | --- | --- | --- |
| Mazzarin CM et al., 2017 | Not reported | Not reported | 64 to 72 years | Older adults with Parkinson's disease | Tai Chi Unspecified, 2 weeks to 12 months | Mobility | Positive | High |
| Chow TH et al., 2018 | Not reported | 1.222 | Not reported | Postmenopausal women, inactive persons, older adults, obese women, and women with osteoarthritis | Tai Chi Unspecified,  ~12 months, up to 185 hours per year | Osteopenia | Positive | High |
| Sherrington C et al., 2019 | Community | 23.407 | 76 years | Older adults with risk of falls | Tai Chi Unspecified, 12 weeks or over | Risk of fall (reduce the rate of fall) | Positive | High |
| Chan JSY, et al., 2019 | Not reported | 3.551 | +60 years | Older adults with mild cognitive impairment | Tai Chi Unspecified, minimal 12 weeks, 3 to 7 times per week, 45 to 60 min. | Motor function | Positive | High |
|  |  |  |  |  |  | Global cognitive function | Positive | High |
| Liu HH et al., 2019 | Not reported | 355 | 40 to 85 years | Older adults with Parkinson's disease | Tai Chi Unspecified, 4 to 24 weeks, 2 to 3 times per week, 60 min. | Motor function | Positive | High |
|  |  |  |  |  |  | Balance |  |  |
|  |  |  |  |  |  | Risk of fall |  |  |
| Ho LYW et al., 2020 | Community | 1.093 | 63.3 to 75.3 years | Older adults with sleep disorders, arthritis, osteoarthritis, and healthy individuals | Tai Chi Unspecified, 4 to 8 weeks, 2 times per week, 1h. | Reduction of fatigue | Positive | High |
| Cai Z et al., 2020 | Not reported | 1.970 | 66 to 82 years | Older adults with cognitive impairment (with or without dementia) | Yang style, 24 weeks, 3 times per week, 30 to 60 min. | Global cognitive function | Positive | High |
| Yang J et al., 2020 | Not reported | 1.061 | 61.5 to 77.8 years | Older adults with mild cognitive impairment | Yang style 24 and 10 forms, 10 weeks to 12 months, 1 to 6 times per week, 30 to 120 min. | Global cognitive function | Positive | High |
|  |  |  |  |  |  | Memory | Positive | High |
|  |  |  |  |  |  | Attention | Positive | High |
|  |  |  |  |  |  | Well being | Positive | High |
| Vanderlinden J et al., 2020 | Community and long-term care centers | Not reported | 64 to 76 years | Healthy older adults | Tai Chi Unspecified, 12 weeks to 6 months, 2 to 3 times per week, 20 to 70 min. | Sleep quality | Positive | High |
| Yang Y et al., 2021 | Community | 1.995 | Not reported | Older adults with low body strength in the lower limbs | Yang and Sun style, minimal 6 weeks, 2 to 4 times per week, 60 min. | Muscle strength | Positive | High |
| Zhao J et al., 2021 | Hospital and long-term care centers | 630 | Not reported | Older adults with physical impairment | Sitting Tai Chi, 12 weeks, 120 to 180 min per week. | Heart rate variability | Positive | High |
|  |  |  |  |  |  | Depression | Positive | High |
|  |  |  |  |  |  | Quality of life | Positive | High |
| Huang CY et al., 2022 | Not reported | 840 | 70-89.5 years | Older adults frailty and with sarcopenia | Yang style, 8 to 48 weeks, 2 to 7 times per week, 30 to 90 min. | Physical function | Positive | High |
|  |  |  |  |  |  | Balance | Positive | High |
|  |  |  |  |  |  | Depression | Positive | High |
|  |  |  |  |  |  | Quality of life | Positive | High |
|  |  |  |  |  |  | Blood pressure | Positive | High |
| Lei H et al., 2022 | Not reported | 996 | Not reported | Older adults with Parkinson's disease | Tai Chi 24 forms, 12 to 24 weeks. | Motor function | Positive | High |
| Song D et al., 2022 | Not reported | 1017 | Not reported | Older adults with mild cognitive impairment | Tai Chi Unspecified, 24 weeks, 3 to 6 times per week, 30 min to 90 min. | Memory | Positive | High |
| Liu X et al., 2022 | Not reported | Not reported | Not reported | Postmenopausal older women | Tai Chi 24 forms, 6 months or over, 3 to 6 times per week, 40 to 60 min. | Patient safety | Positive | High |
|  |  |  |  |  |  | Osteopenia | Positive | High |
| Zhang W et al., 2023 | Community and long-term care centers | Not reported | Not reported | Faller older adults | Yang style, 8 to 24 weeks, 1 to 4 times per week, 20 to 60 min. | Balance | Positive | High |
|  |  |  |  |  |  | Fear of Fall | Positive | High |
|  |  |  |  |  |  | Risk of fall (rate of fall) | Positive | High |
| Xu F et al., 2023 | Not reported | 1.408 | 60 to 70 years | Older adults with Parkinson’s disease, mild cognitive impairment, stroke, sedentary, fear of falling, and/or history of falling | Yang Style, 12 to 24 weeks, 2 times per week, 60 min. | Balance | Positive | High |
| Lyu L et al., 2023 | Community | 1.058 | 52 to 92 years | Older adults with sleep disorders | Tai Chi Unspecified, 2 to 6 months, 3 to 5 times per week, 30 to 60 min. | Sleep quality | Positive | High |
| Chen W et al., 2023 | Not reported | Not reported | Not reported | Older adults' frailty, a non-high risk of falls, history of falls, Parkinson’s disease, and stroke. | Yang and Sun style, 8 to 24 weeks or 12 months, 1 to 3 times per week, 60 min. | Risk of fall | Positive | High |
| Liang T et al., 2023 | Not reported | 1.080 | ~55 years | Not reported clearly | Tai Chi 24 forms, 3 to 24 weeks, 3 to 5 times per week, 30 to 60 min. | Muscle strength | Positive | High |
|  |  |  |  |  |  | Proprioception | Positive | High |
|  |  |  |  |  |  | Balance | Positive | High |
| Kuang X et al., 2024 | Not reported | 1.798 | Not reported | Not reported clearly | Yang style and 24 forms, 1 to 5 times per week, 20 to 90 min. | Anxiety | Positive | High |
|  |  |  |  |  |  | Depression | Positive | High |
| Dong Y et al., 2024 | Not reported | 2.974 | Not reported | Not reported clearly | Tai Chi Unspecified, 4 weeks to 12 months, 1 to 7 times per week, 20 to 90 min. | Anxiety | Positive | High |
|  |  |  |  |  |  | Depression | Positive | High |
| Zhu GC et al., 2024 | Not reported | Not reported | Not reported | Older adults with chronic pain | Not reported | Chronic Pain | Positive | High |
| Su JJ et al., 2024 | Not reported | 2.107 | 62.23 years | Older adults post-stroke | Tai Chi Unspecified, 6 weeks to 6 months, 6 to 168 sessions, 30 to 90 min. | Balance | Positive | High |
|  |  |  |  |  |  | Depression | Positive | High |
|  |  |  |  |  |  | Motor function | Positive | High |
|  |  |  |  |  |  | Activity of Daily Living | Positive | High |
|  |  |  |  |  |  | Quality of life | Positive | High |
| Li Y et al., 2025 | Not reported | 1.877 | Not reported | Older adults with mild cognitive impairment | Tai Chi Unspecified, 7 weeks to 1 year, 1 to 5 times per week, 20 to 90 min. | Physical Function | Positive | High |
|  |  |  |  |  |  | Global cognitive function | Positive | High |
| Dong Y et al., 2025 | Not reported | 2.501 | 60.00 to 81.90 years | Older adults with anxiety and depression | Tai Chi style/form not reported, 12 to 16 weeks, 3 to 4 times per week, 40 to 60 min. | Anxiety | Positive | High |
|  |  |  |  |  | Tai Chi style/form not reported, 24 weeks, 5 to 7 times per week, 40 to 60 min. | Depression | Positive | High |
| Ren Z et al., 2025 | Not reported | 1.077 | Not reported | Not reported clearly | Tai Chi Unspecified, 3 to 12 months, 3 to 7 times per week, 30 to 90 min. | Bone Mineral Density | Positive | High |
| Wan R et al., 2025 | Not reported | 1.838 | 60 to 95 years | Older adults’ frailty and older adults with sarcopenia | Yang style, 8 to 40 weeks, 2 to 7 times per week, 25 and 90 min. | Physical Function | Positive | High |
|  |  |  |  |  |  | Muscle Strength | Positive | High |
| Lei L et al., 2025 | Not reported | 592 | +60 years | Older adults with sleep disorders | Tai Chi Unspecified, 8 to 24 weeks, more than 150 minutes per week | Sleep quality | Positive | High |
| Wang WT et al., 2025 | Community | 1.066 | ~74.1 years | Older adults with mild cognitive impairment | Tai Chi 24 form, Tai Chi 10 form, and Tai Chi 8 form, 12 weeks to 1 year, 2 to 4 times per week, 30 to 60 min. | Executive function | Positive | High |
|  |  |  |  |  |  | Global cognitive function | Positive | High |
| Liu X et al., 2025 | Not reported | 2.489 | Not reported | Older adults with mild cognitive impairment or normal cognitive function | Tai Chi Unspecified, 12 to 48 weeks, 2 to 3 times per week, 50 to 60 min. | Global cognitive function | Positive | High |
